# PRDX1 protects ATM from arsenite-induced proteotoxicity and maintains its stability during DNA damage signaling

**DOI:** 10.18632/oncotarget.28720

**Published:** 2025-05-19

**Authors:** Reem Ali, Mashael Algethami, Amera Sheha, Shatha Alqahtani, Ahmad Altayyar, Ayat Lashen, Emad Rakha, Abdallah Alhaj Sulaiman, Srinivasan Madhusudan, Dindial Ramotar

**Affiliations:** ^1^College of Health and Life Sciences, Hamad Bin Khalifa University, Doha, Qatar; ^2^Naaz Coker Ovarian Cancer Research Centre, Biodiscovery Institute, School of Medicine, University of Nottingham, University Park, Nottingham NG7 3RD, UK; ^3^Department of Pathology, Nottingham University Hospitals, City Hospital Campus, Nottingham NG5 1PB, UK; ^4^Department of Oncology, Nottingham University Hospitals, City Hospital Campus, Nottingham NG5 1PB, UK

**Keywords:** redox signaling, homologous recombination, protein interaction, cell cycle, protein modification

## Abstract

Redox regulation and DNA repair coordination are essential for genomic stability. Peroxiredoxin 1 (PRDX1) is a thiol-dependent peroxidase and a chaperone that protects proteins from excessive oxidation. ATM kinase (Ataxia-Telangiectasia Mutated) and the MRN (MRE11-RAD50-NBS1) complex are DNA damage signaling and repair proteins. We previously showed that cells lacking PRDX1 are sensitive to arsenite, a toxic metal that induces DNA single- and double-strand breaks (DSBs). Herein, we showed that PRDX1 interacts with ATM. PRDX1-deleted cells have reduced ATM, MRE11, and RAD50 protein levels, but not NBS1. In control cells treated with arsenite, we observed γH2AX foci formation due to arsenite-induced DSBs, and not from PRDX1-deleted cells. Arsenite caused profound depletion of ATM in PRDX1-deleted cells, suggesting that PRDX1 protects and stabilizes ATM required to form γH2AX foci. Importantly, arsenite pretreatment of PRDX1-deleted cells caused hypersensitivity to chemotherapeutic agents that generate DSBs. Analysis of a clinical cohort of ovarian cancers treated with platinum chemotherapy revealed that tumours with high PRDX1/high ATM or high PRDX1/high MRE11 expression manifested aggressive phenotypes and poor patient survival. The data suggest that PRDX1 can predict responses to chemotherapy, and targeting PRDX1 could be a viable strategy to improve the efficacy of platinum chemotherapy.

## INTRODUCTION

Peroxiredoxin 1 (PRDX1) is a key member of the PRDX family of thiol-dependent peroxidases widely distributed in tissues [1]. One of the functions of PRDX1 is to prevent the toxic accumulation of hydrogen peroxide (H_2_O_2_) by catalyzing its decomposition using a redox cysteine residue [2, 3]. In addition, PRDX1 is known to act as a chaperone in the oligomeric form to protect key proteins from excessive oxidation [1]. PRDX1 oligomer is known to regulate transcription factors such as p53, nuclear factor Kappa B (NF-κB), and androgen receptor (AR) to prevent oxidative stress-induced death signaling [4]. The crosstalk between PRDX1 and various signaling pathways was previously established including PTEN/AKT signaling and TRAF6 ubiquitin ligase signaling, exerting tumour suppressive roles [5, 6]. Besides PRDX1 role in signaling pathways, it also plays a role in maintaining genomic stability by counteracting DNA damage induced by excessive H_2_O_2_ [7]. A recent study reported that PRDX1 directly binds to the nucleophilic thiol Cys319 residue of the RAD51 protein to protect it from oxidation and maintain its functional role in the homologous recombination DNA repair pathway by facilitating the physical connection between the invading DNA substrate and the homologous DNA template, leading to the formation of the D-loop [7]. This suggests a role for PRDX1 in regulating homologous recombination through redox homeostasis. It appears that PRDX1 might be a central player in diverse physiological processes. In support of this notion, we have recently shown that PRDX1 can interact with the glucose transporter GLUT3 and suppress a cryptic function involved in the uptake of the toxic metalloid arsenite into cells [8]. In the absence of PRDX1, GLUT3 enhances the uptake of arsenite and sensitizes the cells to the metalloid [8]. Consequently, cells devoid of PRDX1 in the presence of GLUT3 are sensitized to the toxic effects of arsenite, although the exact downstream mechanism leading to the toxicity has not been explored.

Arsenite is the water-soluble form of arsenic which is ubiquitously found in the environment and is a class I human carcinogen [9–12]. Exposure to arsenic occurs through drinking water, soil, and food [13]. Arsenic is known to induce reactive oxygen species (ROS) and oxidative stress, which ultimately lead to DNA damage [12]. Arsenic exposure has been linked to longer telomeres in several studies [14–16]. A recent study found an increase in the purine DNA base lesion 8-oxo-2′-deoxyguanosine (8-oxo-dG), a biomarker of DNA oxidative damage, as well as an increase in telomere length, in Bolivian women environmentally exposed to arsenite [17]. Arsenite induces down-regulation of the expression of nucleotide excision repair genes XPA, XPD, and XPF xeroderma pigmentosum A, D, and F through deacetylation of H3K18 acetylation in HaCaT cells [17], suggesting that arsenite can compromise DNA damage repair pathways. Similarly, another study documented that arsenite interferes with homologous recombination, and not the non-homologous end-joining, DNA repair pathway [12]. Low arsenic doses have been used as co-carcinogens to improve the efficacy of other DNA-damaging drugs using cancer cell lines [18]. Clinically, arsenic trioxide is very potent as monotherapy for treating acute promyelocytic leukemia patients that harbor the PML-RARα fusion oncoprotein [19, 20].

Arsenite is believed to generate DNA double-strand breaks that must be repaired by the homologous recombination DNA repair pathway [11, 12, 21]. DNA double-strand breaks (DSBs) are the most deleterious form of DNA damage [22]. It can be induced directly by ionizing radiation or oxidative stress or indirectly through the accumulation of other types of DNA lesions and replication fork stalling [22, 23]. Cellular response to DSB is coordinated by sensors that detect the damaged DNA and activate protein kinases to signal transduction cascades to initiate the repair mechanisms [24]. The ataxia telangiectasia mutated protein (ATM) is a primary signaling kinase in regulating DSBs [23, 25]. ATM exists in a dimeric form in undamaged cells incapable of substrate phosphorylation [26]. Upon DSBs induction, ATM is recruited to the DNA damage sites by the MRE11/RAD50/NBS1 (MRN) complex, which is an early sensor of DSBs [27]. The interaction between ATM and the c-terminal motif in Nbs1 increases ATM accumulation at the DNA damage sites and promotes its autophosphorylation at serine 1981 [28]. ATM autophosphorylation is essential for ATM dissociation into active kinase monomers and interaction with its substrates including the checkpoint protein CHK2 and the histone variant H2AX [27]. ATM phosphorylation of histone H2AX at serine 139 is crucial to DNA damage response leading to the formation of γH2AX foci at sites of DSBs [29]. Thus, MRN-ATM interaction is indispensable for DSB repair signalling [27, 30, 31].

The observations that PRDX1-deleted cells are sensitive to arsenite and that arsenite suppresses the homologous recombination DNA repair pathway, we hypothesize that PRDX1 would regulate this DNA repair pathway. Herein, we provide evidence that PRDX1 interacts with ATM and that PRDX1-depleted cells have lower levels of ATM, and components of the MRN complex compared to the control cells. We show that cells treated with arsenite triggered the formation of γH2AX foci but not in PRDX1-deleted cells with low ATM levels. Examination of the ATM level revealed that arsenite caused its disappearance in the PRDX1-deleted cells, suggesting that PRDX1 protects and stabilizes ATM. Ovarian cancers with high PRDX1/high ATM or high PRDX1/high MRE11 expression showed poor survival and an aggressive phenotype [32]. Thus, targeting PRDX1 should sensitize tumours to DNA-damaging agents.

## RESULTS

### Cells lacking PRDX1 exhibit lower levels of ATM and are hypersensitive to ATM inhibitors

**Figure 1:**
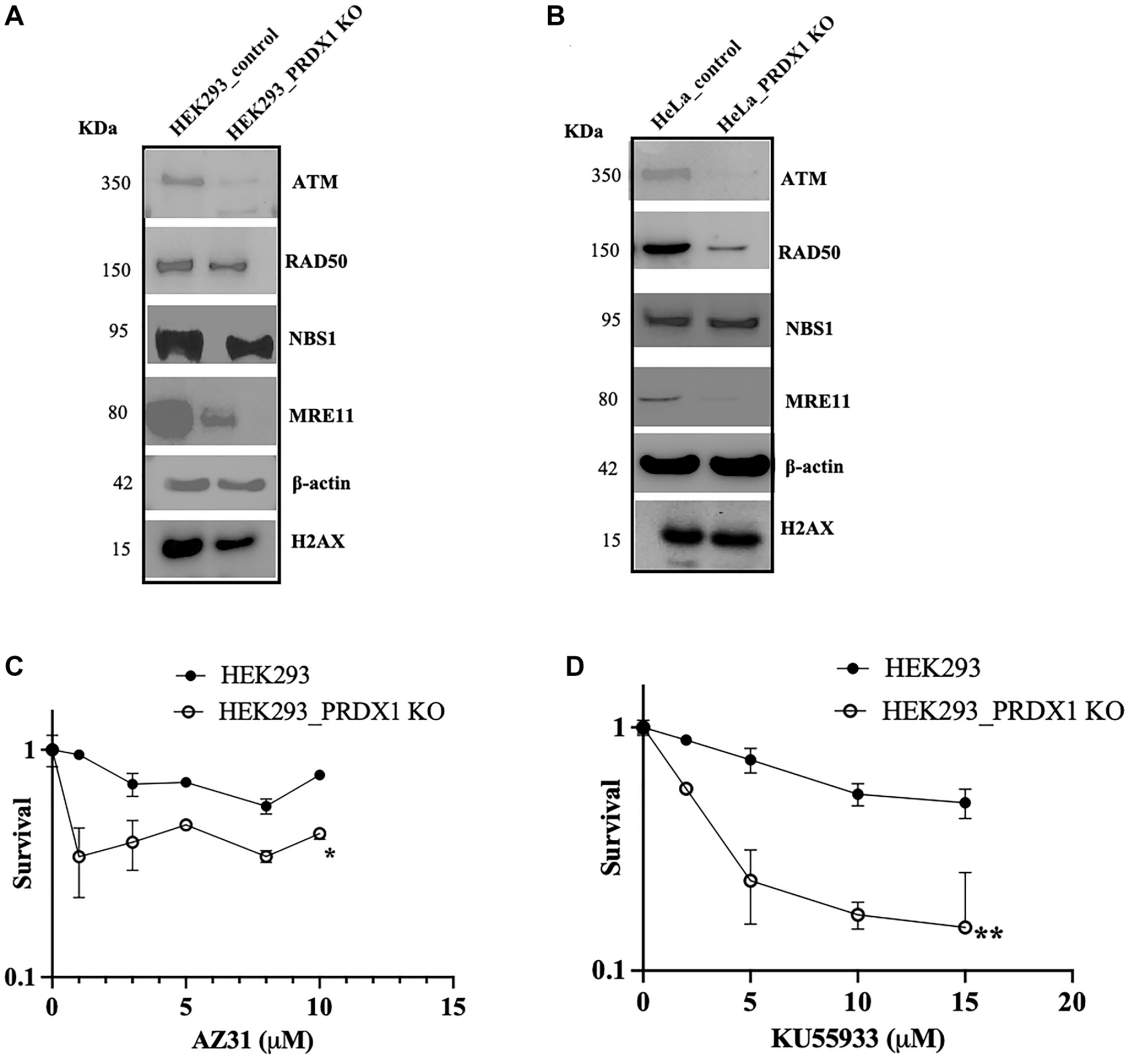
(**A**)Western blot showing ATM, RAD50, NBS1, MRE11 and H2AX protein levels in HEK293 control and HEK293_PRDX1 KO cells. (**B**) Western blot showing ATM, RAD50, NBS1, MRE11 and H2AX protein levels in HeLa control and HeLa_PRDX1 KO cells. (**C**) Clonogenic survival assay showing ATM inhibitor AZ31 sensitivity in HEK293 control and HEK293 PRDX1_KO cells. (**D**) Clonogenic survival assay showing ATM inhibitor KU55933 sensitivity in HEK293 control and HEK293 PRDX1_KO cells. Survival fraction statistical analysis was performed using a two-way ANOVA test. ^*^ *p* < 0.05, ^**^ *p* < 0.01.

We recently demonstrated that PRDX1 knockout cells are sensitive to the metalloid sodium arsenite [8], which has been shown to induce DNA damage including DNA double-strand breaks [12]. Since PRDX1 plays a role in maintaining the function and stability of several proteins e.g., PTEN and ASK1 [33], we examined whether it could modulate proteins of the DNA double-strand break repair pathway in response to arsenite treatment. Thus, we investigated the levels of several components of the homologous recombination DNA repair pathway including ATM, the MRN complex (MRE11, RAD50, NBS1), and H2AX in two different cell types, HEK293 and HeLa, deleted for the PRDX1 by CRISPR-Cas9. We observed diminished levels of ATM, RAD50, MRE11, and H2AX in both HEK293 and HeLa cells deleted for PRDX1, with ATM and MRE11 being more severely affected as compared to the parental cells (Figure 1A, 1B, respectively). Although NBS1 is a component of the MRN complex, it showed no detectable change in the cells deleted for PRDX1 as compared to the parent cells (Figure 1A, 1B), suggesting that PRDX1 may influence the stability of specific proteins involved in the homologous recombination DNA repair pathway and that this pathway could be compromised in PRDX1-deleted cells.

As ATM levels were significantly low in the PRDX1-deleted cells, we tested whether these cells would be sensitive toward the clinically approved ATM small molecule inhibitors AZ31 and KU55933 that lead to DNA damage [34]. As shown in Figure 1C, 1D, the HEK293 PRDX1-deleted cells displayed sensitivity to the ATM inhibitors AZ31 and KU55933, respectively, as compared to the parent cells, suggesting that the PRDX1-deleted cells indeed harbor diminished functional levels of ATM.

### Arsenite induces rapid changes in ATM levels in PRDX1-deleted cells and further sensitizes the cells to AZ31

**Figure 2:**
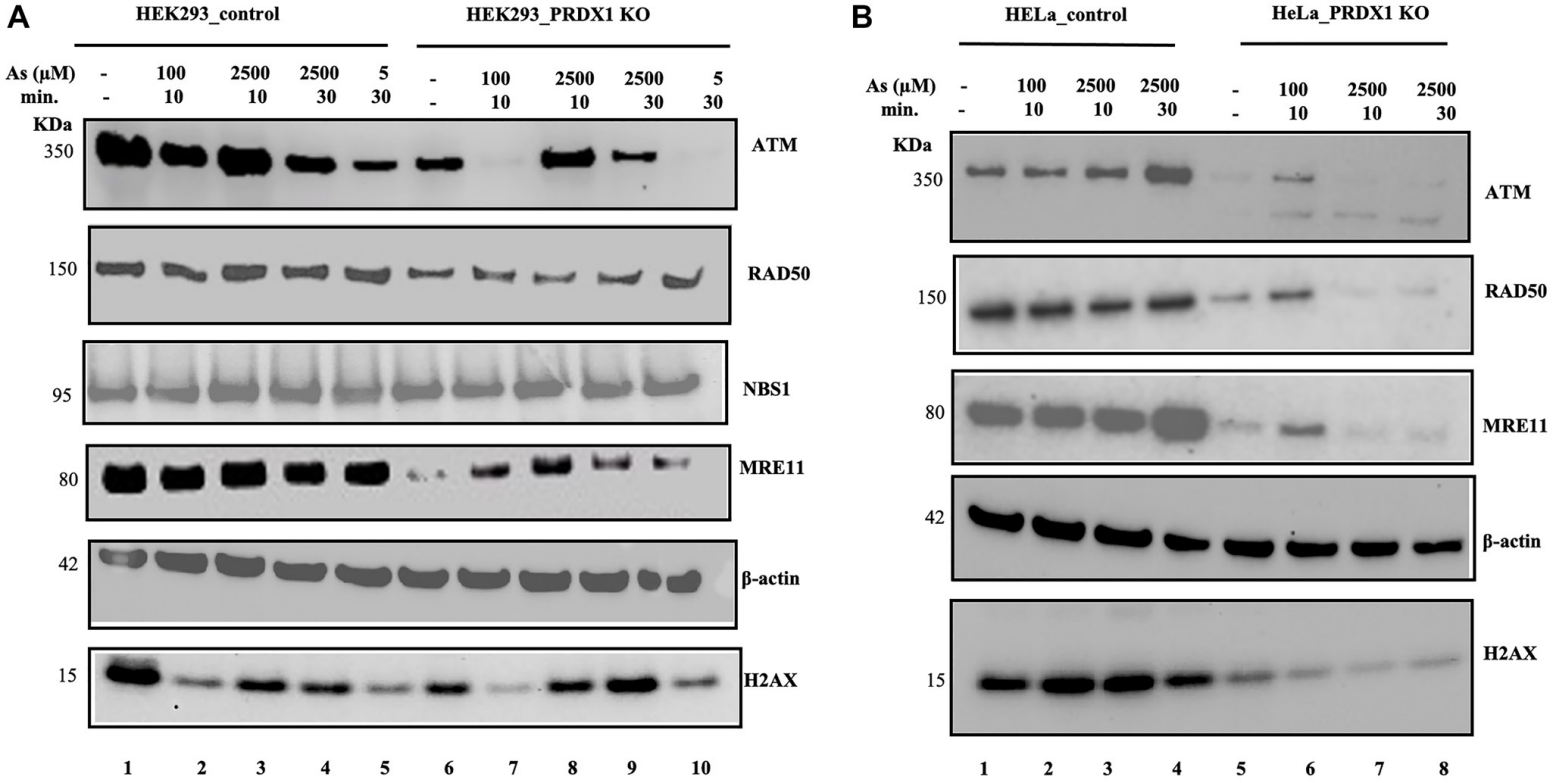
(**A**) Western blot showing ATM, RAD50, NBS1, MRE11, and H2AX protein levels in HEK293 control and HEK293_PRDX1 KO cells treated with arsenite at the indicated doses. (**B**) Western blot showing ATM, RAD50, MRE11, and H2AX protein levels in HeLa control and HeLa_PRDX1 KO cells treated with arsenite at the indicated doses. Briefly, cells were plated overnight, then treated with freshly prepared arsenite doses in PBS. After treatment, cells were incubated with 100 mM N- ethylmaleimide (NEM) for 10 mins on ice, then cells were scrapped and collected by centrifugation. Cell pellets were resuspended in lysis buffer (20 mM Tris- HCL pH 7.4, 100 mM NaCl, 0.5% NP-40, EDTA, 5 mg/ml NEM, and 1X protease and phosphatase inhibitor cocktail) and sonicated. Samples were spun in a microcentrifuge at 13,000 rpm for 10 min at 4°C. Proteins were quantified by micro-BCA and lysates were analyzed by western blot.

Besides damaging the DNA, arsenite is known to modify proteins [35]. There is no evidence whether the inability of cells to repair arsenite-induced DNA lesions is due to inactivated DNA repair proteins or the subsequent loss of the proteins induced by arsenite. As such, we examined the levels of components of the homologous recombination DNA repair pathway in HEK293 and HeLa cells and the isogenic PRDX1-deleted cells in response to different doses of arsenite. As expected, in this independent experiment the PRDX1-deleted HEK293 and Hela cells possessed lower levels of ATM, MRE11, RAD50, and H2AX, but not NBS1, as compared to the respective control cells HEK293 and HeLa (Figure 2A, lane 6 vs. lane 1, and Figure 2B, lane 5 vs. lane 1).

**Figure 3:**
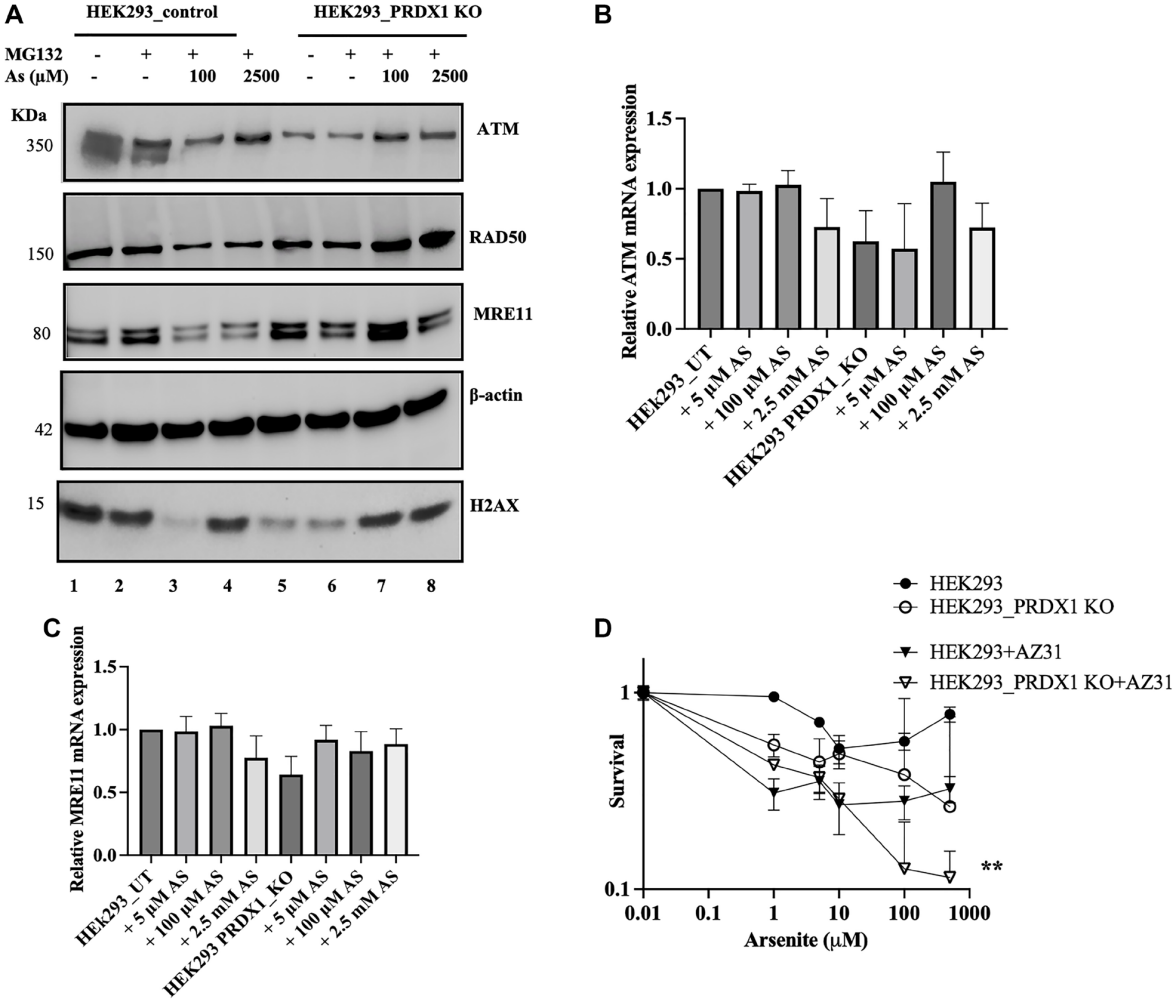
(**A**) Western blot showing ATM, RAD50, MRE11, and H2AX protein levels in HEK293 control and HEK293_PRDX1 KO cells following incubation with MG132 and treatment with arsenite. Cells were plated overnight and then treated with the proteasome inhibitor MG132 (25 μM) for 3 h. Cells were treated with 100 μM or 2.5 mM arsenite for 10 min or left untreated. Cells were washed with 100 mM NEM in PBS and scrapped. Lysates were analyzed by immunoblotting. (**B**) Relative ATM mRNA expression levels by RT-qPCR in HEK293 control and HEK293_PRDX1 KO cells treated with 100 μM or 2.5 mM arsenite for 10 min. (**C**) Relative MRE11 mRNA expression levels by RT-qPCR in HEK293 control and HEK293_PRDX1 KO cells treated with 100 μM or 2.5 mM arsenite for 10 min. GADPH was used for normalization. (**D**) Clonogenic survival assay showing the sensitivity of ATM inhibitor AZ31 plus arsenite combination in HEK293 control and HEK293_PRDX1 KO cells. 500 Cells were plated in 6-well plates overnight and treated with AZ31 (2 μM) for 30 min. Then cells were treated with different arsenite doses in PBS for another 30 min. After incubation cells were topped with fresh media and left to form clones for 10 days. Plates were stained with crystal violet Methanol mixture. Survival fraction statistical analysis was performed using a two-way ANOVA test, ^**^ *p* < 0.01.

Upon treatment with arsenite, the ATM, MRE11, and H2AX proteins showed cell type-specific responses in the PRDX1-deleted cells. For example, the HEK293 PRDX1-deleted cells showed a complete disappearance of ATM following a low-dose treatment (100 μM for 10 min) with arsenite compared to the untreated cells (Figure 2A, lane 7 vs. lane 6). The disappearance of ATM triggered by the low-dose arsenite was blocked by pretreating the PRDX1-deleted HEK293 cells with the 26S protease inhibitor MG132, suggesting the involvement of proteolysis in the disappearance of ATM (Figure 3A, lane 7 vs. Figure 2A, lane 7). However, the effect of MG132 was apparent in the PRDX1-deleted cells and not in the control cells (Figure 3A, lane 7 vs. 3).

We checked whether other components of the homologous recombination DNA repair pathway were similarly affected as ATM upon treatment with arsenite. As shown in Figure 2A, H2AX was also similarly diminished as ATM upon exposure of the HEK293 PRDX1-deleted cells to the low dose of arsenite (Figure 2A, lane 7 vs. 6), although the effect on MRE11 and RAD50, and not NBS1, appeared to be variable with the dose of arsenite. As observed for ATM, these PRDX1-deleted cells also possess increased levels, particularly MRE11 and H2AX when exposed to the high dose of arsenite (2500 μM for 10 min) (Figure 2A, lane 8 vs. 7). It is possible that the high dose of arsenite may induce the expression of ATM, MRE11, and H2AX or impede the process required to turn over these proteins.

To eliminate the possibility that the variation in the protein levels in response to arsenite could be associated with alteration in gene expression, total RNA was extracted from the control and PRDX1-deleted cells untreated and treated with the indicated doses of arsenite and checked for ATM and MRE11 mRNA levels. qPCR analysis revealed no significant effect on ATM or the MRE11 gene expression following treatment with a range of arsenite concentrations in the control or the PRDX-deleted cells (Figure 3B, 3C). This analysis contrasts the protein expression pattern seen particularly in the PRDX1-deleted cells (Figure 2A), where the protein levels varied in response to arsenite treatment. The data suggest that PRDX1 is not involved in regulating the gene expression of components of the recombinational DNA repair pathway, and instead, appears to protect the proteins from the toxic effects of arsenite.

It is noteworthy that in HeLa cells, PRDX1 also provided similar protective effects to the components of the recombinational DNA repair pathway, although the disappearance of the proteins appeared to be different with the low- and high-dose of arsenite treatments in the HeLa PRDX1-deleted cells as compared to the HEK293 PRDX1-deleted cells (Figure 2B). Moreover, a prominent ATM fragment was present in the HeLa PRDX1-deleted cells following arsenite treatment (Figure 2B, lanes 6 to 8), which was not detectable in the HEK293 PRDX1-deleted cells. Nonetheless, the data support the notion that PRDX1 protects ATM and other proteins of the homologous recombination DNA repair pathway from arsenite-induced degradation.

Based on the above findings, we checked whether inhibiting the residual level of ATM kinase activity with AZD31 in the HEK293 PRDX1-deleted cells would further sensitize these cells to arsenite. The data revealed that HEK293 PRDX1-deleted cells pretreated with the ATM inhibitor AZ31 (2 μM for 30 mins) followed by increasing concentrations of arsenite showed a significant decrease in survival as compared to the same cells treated with arsenite alone (Figure 3D). In contrast, the effect was less profound in the PRDX1-proficient HEK293 cells (Figure 3D). It would appear that the sharp reduction in the viability of the PRDX1-deleted cells is due to the complete loss of ATM function caused by the combination of AZ31 and arsenite treatment.

### PRDX1 is required for ATM-dependent phosphorylation of H2AX in response to arsenite

**Figure 4:**
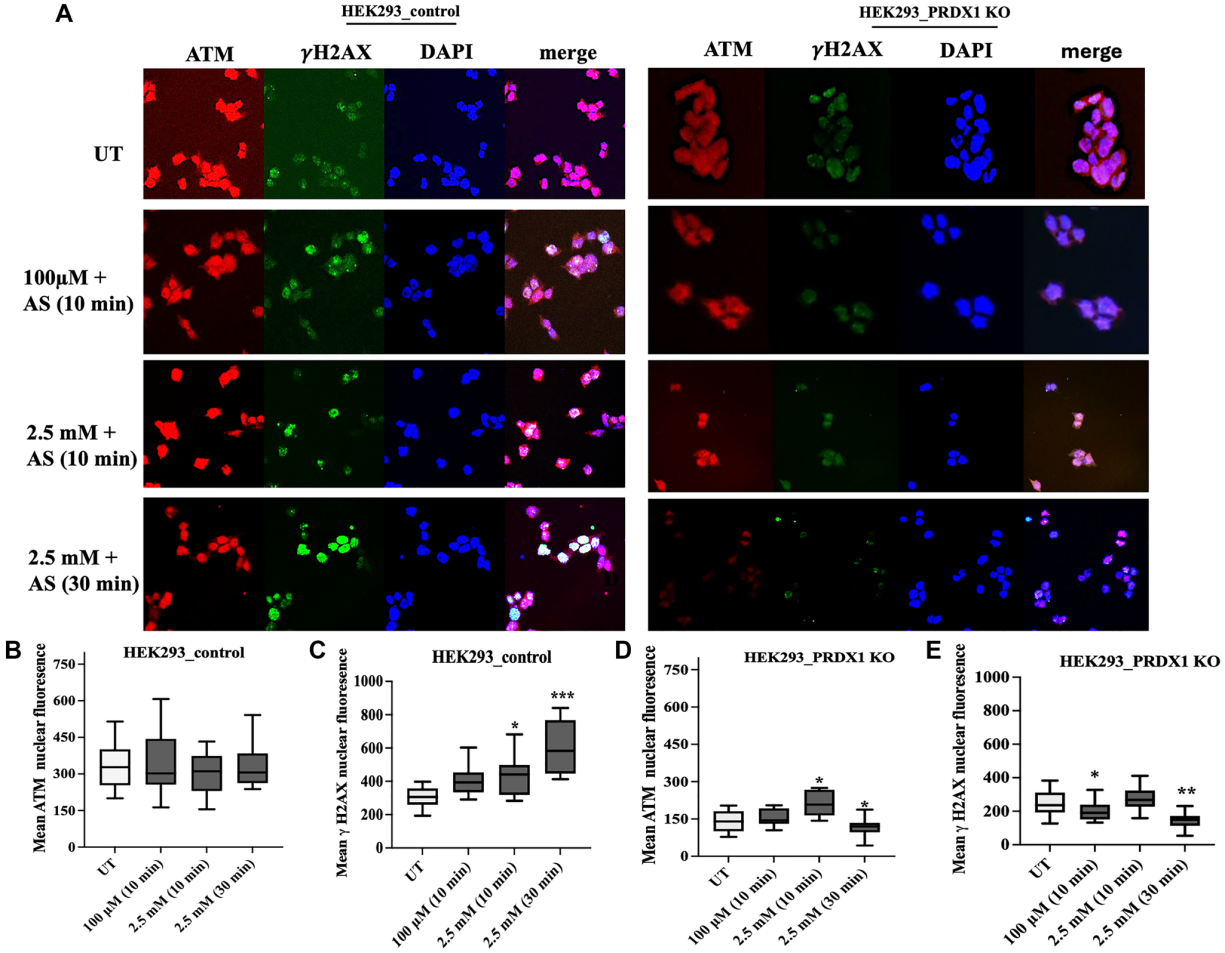
(**A**) Representative photomicrographic images showing HEK293 control and HEK293 PRDX1 KO cells treated with the indicated doses of arsenite. (**B**, **C**) Quantification of ATM and γH2AX nuclear fluorescence, respectively, in control cells by ImageJ Software. (**D**, **E**) Quantification of ATM and γH2AX nuclear fluorescence, respectively, in HEK293_PRDX1 KO cells by ImageJ Software. Statistical analysis was performed using one-way ANOVA. The error bars represent the mean ± SD. ^*^ *p* < 0.05, ^**^ *p* < 0.01, ^***^ *p* < 0.001.

ATM has been shown to phosphorylate H2AX at serine 139 (γH2AX) in response to DNA damage [29]. Since the ATM level in the PRDX1-deleted cells was affected by arsenite exposure (Figure 2), we next checked whether this treatment would interfere with the ability of ATM to activate the DNA damage response by examining γH2AX levels. To carry out this experiment, we exposed the HEK293 control and HEK293 PRDX1-deleted cells to different doses of arsenite, as in Figure 2, and monitored γH2AX and ATM nuclear fluorescence by confocal microscopy. In the absence of arsenite treatment (UT), there was a very low basal level of γH2AX detected by anti-γH2AX antibodies in both the HEK293 control and PRDX1-deleted cells (Figure 4A, and see quantification 4C, 4E). Upon treatment with the low dose of arsenite (100 μM for 10 mins), there was a sharp elevation of the γH2AX signal in the control cells, but not in the case of the PRDX1-deleted cells (Figure 4A, and quantification 4C, 4E). It is noteworthy that under the same low dose of arsenite treatment, the PRDX1-deleted cells exhibited a significant loss of ATM, as well as H2AX (Figure 2, lane 7 vs. lane 6), which may explain the lack of detectable γH2AX signal (Figure 4A, and see quantification 4B, 4D). Interestingly, even though ATM accumulated in the PRDX1-deleted cells treated with a high dose of arsenite (2500 μM for 10 mins), there was no phosphorylation of γH2AX, as compared to the control HEK293 cells treated with the same high dose of arsenite (Figure 4). A simple interpretation of these data is that ATM appears to be inactivated by arsenite in the absence of PRDX1. Thus, under these conditions, ATM cannot mediate the phosphorylation of H2AX to initiate DNA repair raising the possibility that PRDX1 might interact with ATM (see below).

**Figure 5:**
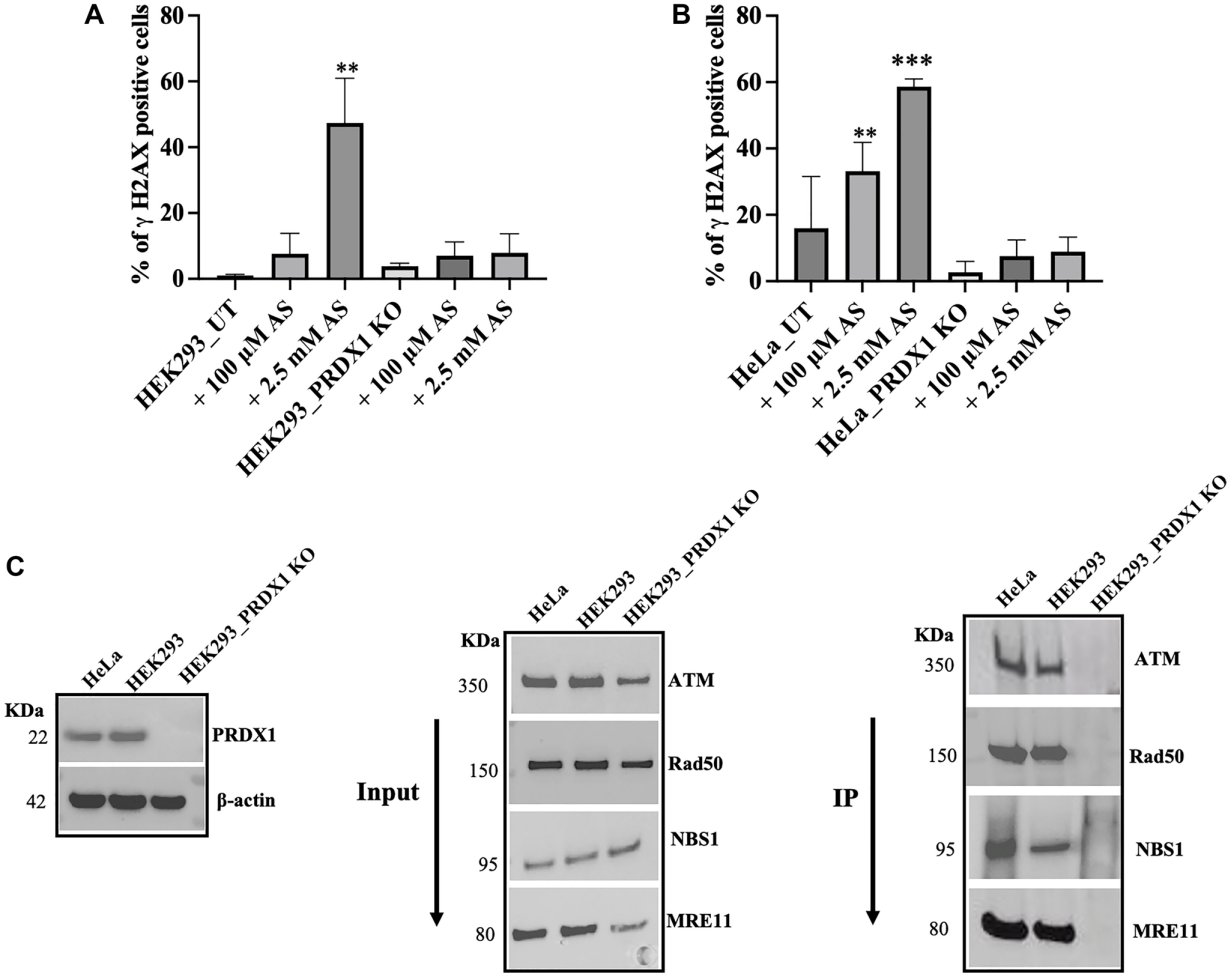
(**A**) Quantification of γH2AX positive cells by flow cytometry in HEK293 control and HEK293 PRDX1 KO cells treated with 100 μM or 2.5 mM arsenite for 30 min. (**B**) Quantification of γH2AX positive cells by flow cytometry in HeLa control and HeLa PRDX1 KO cells treated with 100 μM or 2.5 mM arsenite for 30 min. Cells were plated in 6-well plates overnight and treated with arsenite in PBS (100 μM or 2.5 mM arsenite for 30 min) then cells were washed and left to recover in fresh media for 16 h. Cells were fixed in 70% ethanol for 30 min and stained with propidium iodide and FITC -γH2AX. Cells were analyzed by flow cytometry, and data analysis was performed in FlowJo software. (**C**) Co-immunoprecipitation shows the interaction between PRDX1 and ATM, MRN complex in HeLa and HEK293 control cells. HEK293 PRDX1 KO cells were used as a negative control. Statistical analysis was performed using one-way ANOVA. The error bars represent the mean ± SD., ^**^ *p* < 0.01, ^***^ *p* < 0.001.

To confirm that PRDX1 is required to mediate the phosphorylation of H2AX in response to arsenite-induced DNA damage exposure, we analyzed γH2AX foci formation using flow cytometry. For this experiment, the cells were challenged with arsenite with the indicated doses (either 100 μM or 2500 μM for 30 mins), washed, and allowed to recover in fresh media for 16 h followed by flow cytometry. We observed an induction of γH2AX foci in both the HEK293 and the HeLa control cells treated with arsenite at either dose, however, the effect was more pronounced with the higher dose of arsenite (Figure 5A, 5B, and Supplementary Figure 1). In contrast, γH2AX foci formation was extremely weak in the PRDX1-deleted cells (Figure 5A, 5B, and Supplementary Figure 1). The result indicates that PRDX1 may be required to activate ATM that triggers the phosphorylation of H2AX at arsenite-induced DNA strand breaks.

### PRDX1 interacts with ATM and the MRN complex

PRDX1 is known to act as a chaperone to prevent oxidation and subsequent destruction of several signaling proteins [3, 8]. The observation that the levels of ATM, MRE11, RAD50, and H2AX were significantly reduced in the PRDX1-deleted cells, suggests that PRDX1 might interact with components of the homologous recombination DNA repair pathway. To check this, we prepared total extracts from HeLa and HEK293 controls and the PRDX1-deleted cells and performed a co-immunoprecipitation assay using anti-PRDX1 antibody (Figure 5C). The anti-PRDX1 antibody pulled down the ATM, MRE11, RAD50, and NBS1 proteins from extracts prepared from either the HeLa or HEK293 control cells (Figure 5C). In contrast, these homologous recombination proteins were not efficiently pulled down from extracts derived from the PRDX1-deleted cells (Figure 5C), indicating that immunoprecipitating the homologous recombination complex depends on PRDX1. It is noteworthy that since PRDX1 has been shown to bind to DNA [36], the cell lysates were pre-treated with DNaseI for 30 min before subjecting the samples to co-immunoprecipitation with the PRDX1 antibody. This step was introduced to eliminate the possibility that PRDX1 was pulling down ATM, MRE11, RAD50, and the NBS1 proteins due to their ability to associate with DNA. Based on these findings, it would appear that PRDX1 interacts with ATM and the MRN protein complex and maintain their stability under normal aerobic conditions and when the cells are exposed to stress conditions.

### PRDX1-deleted cells are defective in cell cycle arrest in response to arsenite

**Figure 6:**
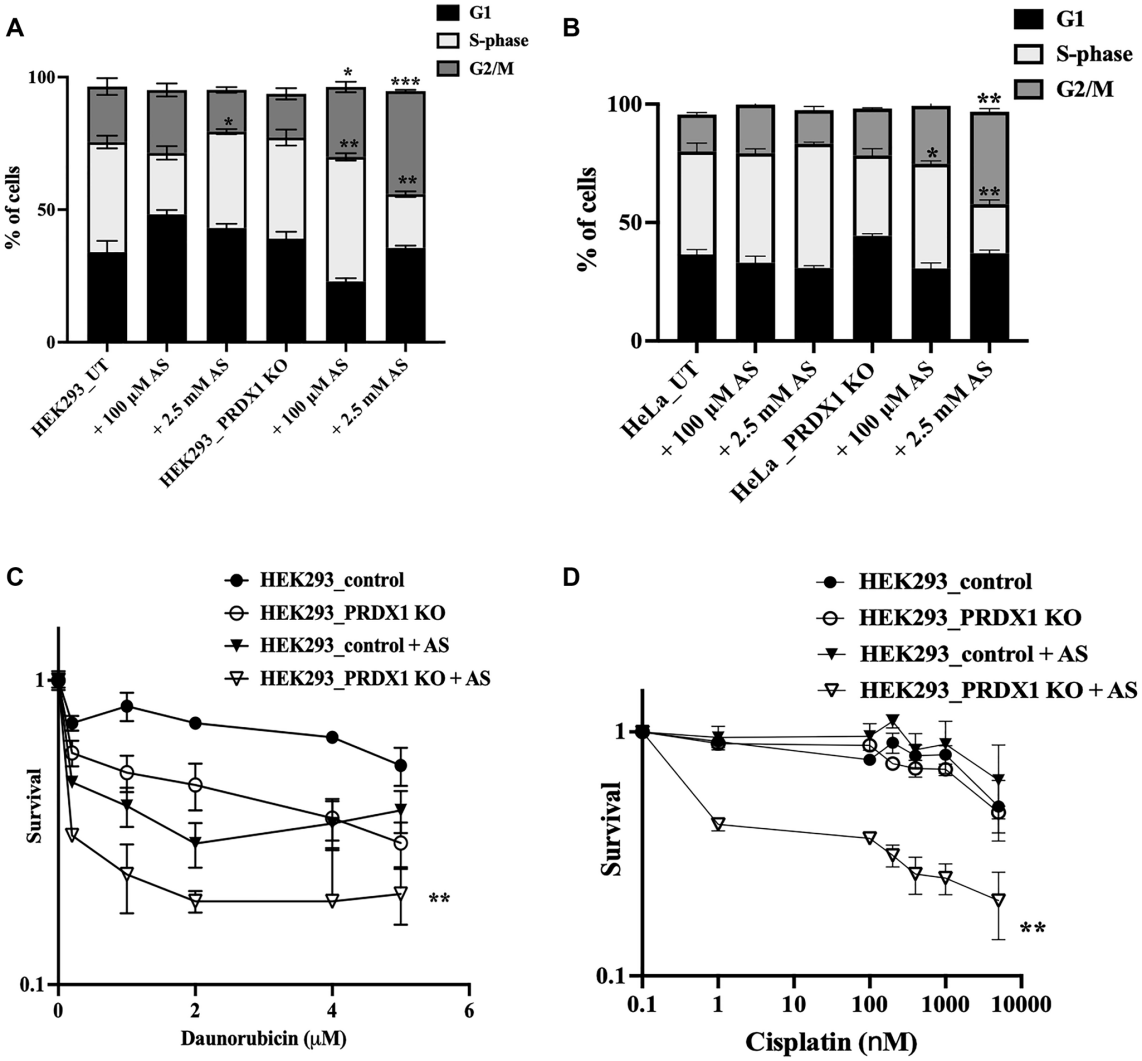
(**A**) Cell cycle analysis by flow cytometry software in HEK293 control and HEK293 PRDX1 KO cells treated with arsenite. (**B**) Cell cycle analysis by flow cytometry software in HeLa control and HeLa PRDX1 KO cells treated with arsenite. Cells were plated in 6-well plates overnight, the following day cells were treated with arsenite in PBS (100 μM or 2.5 mM arsenite for 30 min). Cells were washed and left to recover in fresh media for 16 h. Cells were fixed in 70% ethanol for 30 min and stained with propidium iodide and FITC -γH2AX. Cells were analyzed by flow cytometry and data analysis was performed in FlowJo software. (**C**) Daunorubicin sensitivity in HEK293 control and HEK293 PRDX1 KO cells pre-treated with arsenite followed by clonogenic survival assay. (**D**) Cisplatin sensitivity in HEK293 control and HEK293 PRDX1 KO cells pre-treated with arsenite followed by clonogenic survival assay. Cell cycle statistical analysis was performed using two-way ANOVA. The error bars represent the mean ± SD., ^*^ *p* < 0.05, ^**^ *p* < 0.01, ^***^ *p* < 0.001. Survival statistical analysis was performed using two-way ANOVA, ^**^ *p* < 0.01.

ATM performs multiple roles including the activation of the checkpoint pathways in response to genotoxic agents allowing cells to efficiently repair damaged DNA [23]. We checked whether arsenite-induced DNA damage would elicit a checkpoint response requiring the function of ATM in control and PRDX1-deleted cells. The experiment was conducted as in Figure 4 above and following the arsenite treatment, cells were washed, allowed to recover in fresh media for 16 h, and subjected to flow cytometry analysis. As shown in Figure 6, the untreated PRDX1-deleted cells showed a modest delay in the G1/S phase (Figure 6A, 6B). Upon arsenite treatment (100 μM for 30 mins), we observed an accumulation of cells in the G1/S phase in the HEK293 control cells, while significantly fewer cells accumulated in the G1/S phase in the PRDX1-deleted cells, and instead, these cells accumulated in the S-phase (Figure 6A). The data are consistent with the requirement for ATM to operate efficiently and trigger the G1/S phase arrest. It is noteworthy that increasing the dose of arsenite (2500 μM for 30 mins) caused substantial G2/M arrest in the PRDX1-deleted cells (Figure 6A, 6B and Supplementary Figure 2), suggesting that these cells are accumulating unrepaired DNA double-strand breaks.

### Arsenite pre-treatment sensitizes PRDX1-deleted cells to agents that induce DNA double-strand breaks

PRDX1-deficient cells have lower levels of ATM, MRE11, and RAD50 and these protein levels were further diminished by arsenite treatment (Figure 2). We anticipated that pre-treatment of PRDX1-deleted cells with low dose of arsenite would hypersensitize these cells to DNA damaging agents. To assess this, we pre-treated the HEK293 control and the PRDX1-deleted cells with 5 μM arsenite for 30 min, followed by washing the cells to remove the arsenite, and then subjected the washed cells to either treatment with daunorubicin or cisplatin (Figure 6C, 6D). The PRDX1-deleted cells were more sensitive to daunorubicin than cisplatin when compared to the control cells, suggesting that the PRDX1-deleted cells exhibit a reduced capacity to repair daunorubicin-induced DNA double-strand breaks [37] (Figure 6C vs. 6D). However, if the cells were pretreated with arsenite followed by treatment with either daunorubicin or cisplatin, the PRDX1-deleted cells displayed significantly higher sensitivity to both daunorubicin and cisplatin compared to the control cells (Figure 6C, 6D, respectively). Together the data provide evidence that PRDX1 regulates the homologous recombination DNA repair pathway through interaction with ATM, the MRN complex, and H2AX to maintain their functionality.

### Clinicopathological significance of PRDX1, ATM, and MRE11 co-expression in human ovarian cancers

**Figure 7:**
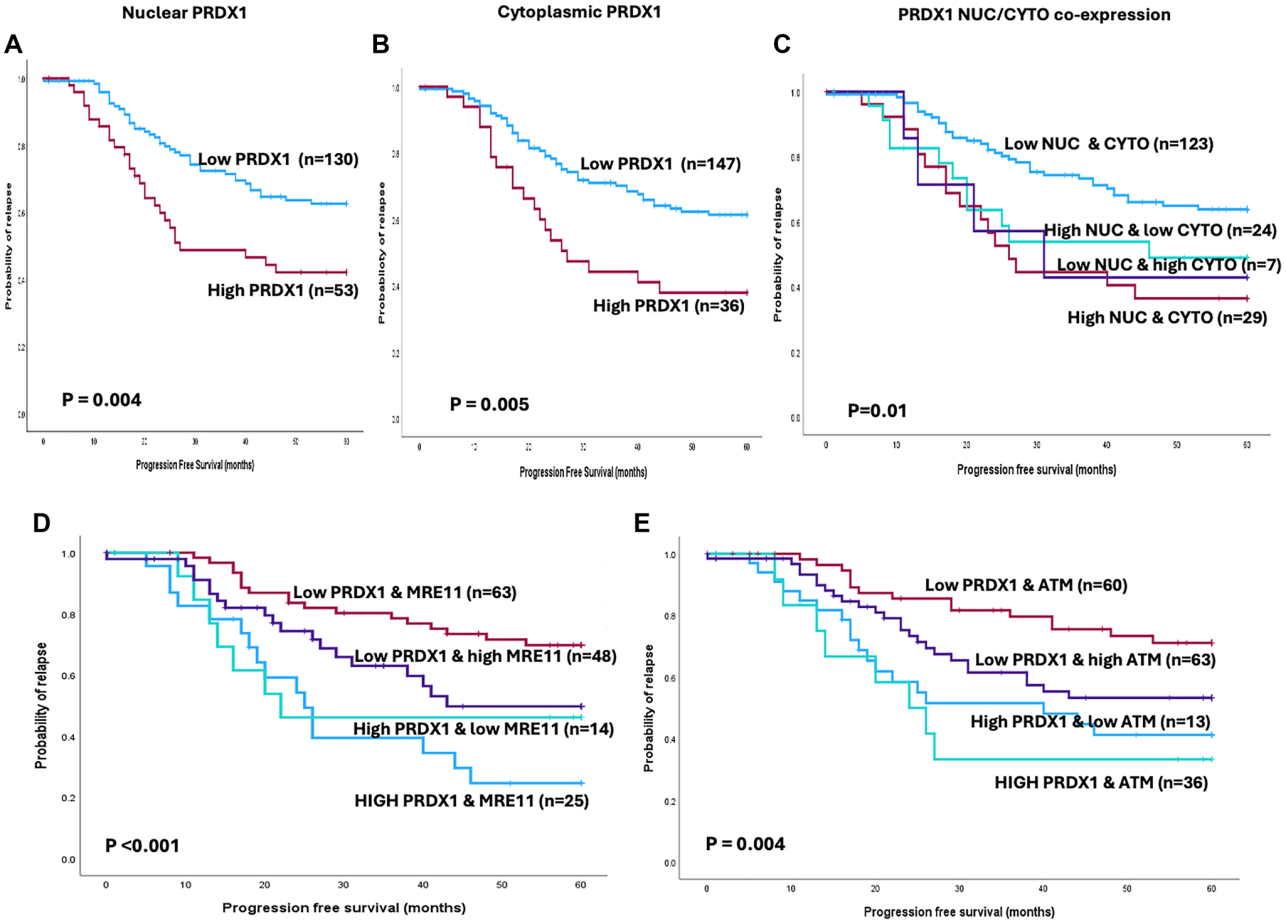
Kaplan-Meier survival analysis shows ovarian cancer progression-free survival and (**A**) PRDX1 nuclear expression. (**B**) PRDX1 cytoplasmic expression and (**C**) PRDX1 nuclear/cytoplasmic expression. (**D**) Kaplan-Meier survival analysis shows ovarian cancer progression-free survival and PRDX1/MRE11 co-expression. (**E**) Kaplan-Meier survival analysis shows ovarian cancer progression-free survival and PRDX1/ATM co-expression.

The expression of PRDX1, ATM, and MRE11 were investigated using Tissue MicroArrays (TMA) of 331 consecutive ovarian epithelial cancer cases treated at Nottingham University Hospitals (NUH) between 1997 and 2010. Not all cores within the TMA were suitable for immunohistochemical (IHC) analysis due to missing cores or the absence of tumour cells. As a result, a total of 183 tumours were suitable for PRDX1 IHC analysis. PRDX1 staining was observed in both the nuclear and cytoplasmic compartments (Figure 7 and Supplementary Figure 3). Of these tumours, 130 out of the 183 were low for PRDX1 nuclear expression, and the other 53 showed high expression (Figure 7A). In the case of cytoplasmic expression, 147 out of the 183 tumours were low for PRDX1 expression and 36 tumours exhibited high expression (Figure 7B). The high PRDX1 nuclear and cytoplasmic expression was associated with adverse clinical outcomes with shorter progression-free survival (PFS) (*p* = 0.004) and (*p* = 0.005) (Figure 7A, 7B) and poor overall survival (OS) (*p* = 0.06) and (*p* = 0.08) (Supplementary Figure 4A, 4B). The clinical data suggest that high PRDX1 is a predictor of response to platinum chemotherapy and poor prognosis in ovarian cancer. When the nuclear and cytoplasmic expression of PRDX1 were combined, the high expression from both compartments remained associated with poor PFS and worse patient survival outcomes (Figure 7C and Supplementary Figure 4C).

As shown above, PRDX1 interacted with the MRN complex, and we previously showed that high MRE11 expression in ovarian tumors is linked to an aggressive phenotype and predicts platinum resistance [38]. We conducted PRDX1/MRE11 and PRDX1/ATM co-expression analysis (Figure 7D, 7E). Tumours with low PRDX1/MRE11 co-expression have favorable PFS (Figure 7D) as well as overall survival (OS) (Supplementary Figure 4D) compared to tumours with high PRDX1/MRE11. Similarly, low PRDX1/ATM co-expression was associated with better PFS (Figure 7E) and overall survival (OS) (Supplementary Figure 4E) compared to tumours with high PRDX1/high ATM expression. Together the data support the hypothesis that PRDX1 interaction with ATM and the MRN components could influence patients’ prognosis in ovarian cancers.

## DISCUSSION

We previously showed that PRDX1-deficient cells are hypersensitive to the metalloid sodium arsenite [8]. Arsenite exerts its toxicity on cells by acting as a potent DNA-damage inducer creating both single- and double-strand breaks that are repaired by the homologous recombinational DNA repair pathway [39]. In addition, arsenite can destabilize proteins by forming covalent bonds with the cysteine residues of proteins [40]. Importantly, arsenite has been established as a therapeutic agent for the treatment of acute promyelocytic leukemia [19, 20, 41, 42]. In this study, we investigated the role of PRDX1 in arsenite-induced DNA damage response, particularly focusing on its interaction with the ATM and MRN complex and its influence on the homologous recombination DNA repair pathway. The MRN complex is an early sensor for DNA double-strand breaks [43] and plays a crucial role in activating the homologous recombination DNA repair pathway by recruiting ATM, which in turn phosphorylates several components to promote cell cycle arrest and allow efficient DNA repair [27, 43, 44]. Herein, we show that PRDX1-deleted cells, in comparison to the control cells, displayed lower levels of key components of the homologous recombinational DNA repair pathway and these proteins include ATM, MRN, and H2AX. Strikingly, the levels of these proteins were further reduced and appeared to be rapidly degraded in the PRDX1-deleted cells challenged with arsenite. Thus, the selective toxicity of PRDX1-deficient cells towards arsenite can be explained by the inability of these cells to repair arsenite-induced DNA lesions by the homologous recombinational DNA repair pathway. Consistent with these observations, the low level of ATM in the PRDX1-deleted cells sensitized the cells to ATM inhibitors. These PRDX1-deleted cells were synergistically sensitized by the pretreatment with the ATM inhibitor followed by the subsequent treatment with arsenite underscoring the importance of preserving the homeostatic levels of ATM by PRDX1. Our results provide compelling evidence that PRDX1 plays a crucial role in maintaining the stability and function of ATM and the MRN complex, and genetic defects in PRDX1 are expected to affect cellular responses to DNA damage and influence cell survival following exposure to genotoxic agents.

It is well established that PRDX1-deleted cancer cell lines accumulate high levels of reactive oxygen species (ROS), which have the propensity to oxidize proteins, as well as create oxidative DNA lesions [4]. PRDX1 can act as a chaperone and protect proteins from ROS-induced inactivation and degradation [45–47]. It can interact with the phosphatase PTEN and prevent oxidation of either Cys71 or Cys124, which could result in the formation of a disulfide bond and consequently inhibit the phosphatase activity [48]. Besides its chaperone function, PRDX1 is also involved in redox relays, whereupon scavenging H_2_O_2_ the peroxidatic cysteine residue (Cys52) becomes oxidized to sulfenic acid (Cys52-SOH), and instead of forming a disulfide bond with the resolving cysteine173 of another molecule of PRDX1 for regeneration to the reduced state, it can react with the nucleophilic thiol group of another protein that is susceptible to oxidation [33]. A classic example involves the oxidation of the MAP3K apoptosis signal-regulating kinase 1 (ASK1) by PRDX1, which triggers the autophosphorylation of ASK1 leading to the activation of its downstream targets such as c-Jun and the p38 MAP kinase pathways [33, 49–51]. Similarly, another peroxiredoxin family member PRDX2 in its oxidized form can directly interact with STAT3 to form a disulfide-linked conjugate blocking STAT3 transcriptional activation function at serum-induced promoter [52]. Considering that PRDX1 is involved in redox-relay, the association of PRDX1 with ATM is due to the formation of PRDX1-ATM disulfide-linked conjugates with cysteines of ATM such as the cysteine Cys2991 that is susceptible to oxidation [52–54]. We believe that this relay by PRDX1-ATM may serve to either stabilize ATM and prevent its degradation or activate ATM function or both. In support of these possibilities, cells lacking PRDX1 possess a lower level of ATM which rapidly disappeared in response to low doses of arsenite. In addition, cells lacking PRDX1 could not activate ATM-dependent phosphorylation of H2AX, which is required to recruit components for efficient repair of DNA double-strand breaks by the homologous recombination repair pathway. It seems that in the absence of PRDX1, ATM is left exposed to be modified and inactivated by arsenite followed by its subsequent degradation.

There are many examples where trivalent arsenicals can bind to the sulfhydryl group of cysteine residues of proteins causing either conformational changes, protein aggregation, destabilization, or enzymatic inactivation of the proteins [40]. Earlier work revealed that arsenite binds directly to the Cys3HisCys4 amino acid motif of the RING finger domains of RNF20-RNF40 heterodimer [55]. RNF20-RNF40 heterodimer signals histone H2B lysine 120 (K120) for monoubiquitination which is crucial for DNA double-strand break repair [55]. Thus, arsenite binding to RNF20-RNF40 impairs DSB repair through inhibition of histone H2B ubiquitination [56, 57]. A more recent study by Dong et al., (2022) used a chemoproteomic approach to selectively capture proteins from lysates of HEK293T cells that are bound to biotinylated arsenite (37). The approach led to the identification of 409 proteins and 51 of these are potential arsenic-binding proteins that include molecular chaperones such as the heat shock proteins HSP1 and HSPA4 (37). Amongst the 409 proteins that bind biotinylated arsenite, ATM was not present, but interestingly the list contains members of the PRDXs family including PRDX1 and a component of the homologous recombination DNA repair pathway, namely RPA, involved in protecting single-stranded DNA for the recruitment of the RAD51 recombinase (see below). Although there is no simple approach for detecting arsenite-bound proteins by mass spectrometry, the method used by Dong et al., (2022) that incorporated the biotin moiety to create the biotinylated-arsenite would likely cause a steric hindrance and prevent arsenite from conjugating with cysteines for example in the ATM and p53 proteins, as the latter is known to bind arsenite [58]. Nonetheless, the report by Dong et al., uncovered that the subunits PSMB6, PSMA6, and PSMD2 of the 20S proteasome are directly binding to arsenic [40]. Our observation that PRDX1-deleted cells treated with a low dose of arsenite triggered the rapid degradation of ATM, but caused its accumulation if these cells were treated with a high dose could be explained by the inactivation of the proteasomal complex at higher concentrations of arsenite. Therefore, it is likely that proteases are targeted by arsenite in PRDX1-deficient cells.

It has been shown that *prdx1*^−/−^ null mice do not display embryonic lethality, but these mice develop various abnormalities that include a high prevalence of lymphomas and liver carcinomas [59]. Similar to cell lines devoid of PRDX1, tissues from *prdx1*^−/−^ null mice display a high level of ROS associated with a significant increase of ROS-induced oxidatively damaged DNA bases that were detected by liquid- and gas-chromatography/mass spectroscopy [60]. These damaged DNA bases such as the hydroxylated purines 8-hydroxy-2′-deoxyadenosine and 8-hydroxy-2′-deoxyguanosine, as well as the cyclic nucleosides (5′ R, S)-cyclo-2′-deoxyadenosine, and (5′ R, S)-cyclo-2′-deoxyguanosine, are capable of blocking transcription and replication [60]. These toxic lesions, if remain unrepaired, can stall and collapse the movement of DNA replication forks causing DNA double-strand breaks that lead to chromosomal rearrangements and translocations and consequently the development of diseases such as cancers [59]. Thus, the high incidence of lymphomas and liver carcinomas in the *prdx1*^−/−^ null mice might be explained by the necessity of PRDX1 to preserve the functionality of ATM required to repair DNA double-strand breaks [59]. A separate study by Skoko et al. (2022) highlighted the crucial role of PRDX1 in maintaining the functionality of the homologous recombination DNA repair pathway in response to DNA damage [7]. The authors showed that PRDX1 is required to protect the cysteine residue Cys319 of RAD51 from oxidation by maintaining its reduced state and the protein remains functionally active [7]. RAD51 is a recombinase that displaces RPA on single-stranded DNA to form a RAD51-coated DNA filament for invasion into the homologous duplex DNA to initiate repair, and this biochemical reaction depends on the reduced form of RAD51 [7]. PRDX1 depletion sensitized cells to ionizing radiation as these cells are unable to reduce RAD51 to form RAD51 foci at radiation-induced DNA strand breaks and therefore impede the homologous recombination repair pathway [7]. In this scenario, reduced PRDX1 and not oxidized PRDX1 (PRDX1-Cys52-SOH) may be required to maintain cysteine residues of ATM in the reduced state, which are susceptible to oxidation.

Low PRDX1 protein and mRNA expression have been linked to improved survival and better prognosis in gastric cancer [61, 62], breast cancer [3, 63], and hepatocellular carcinoma [64], which was previously attributed only to the role of PRDX1 in redox signaling [3]. In our ovarian cancer cohort, high PRDX1 was associated with poor survival and worse outcomes for ovarian cancer patients in line with findings from other clinical studies [3, 61, 65]. However, the role of PRDX1 in regulating homologous recombination pathway response is a likely explanation for the poor prognosis of high-expressing PRDX1 tumours. Chemotherapeutic drugs generate DNA adducts that get converted to double-strand breaks during replication [66, 67]. Upregulation of homologous recombination pathway genes was previously linked to chemotherapy resistance and poor prognosis [68, 69]. It is reasonable to postulate that high PRDX1 expression will maintain optimal levels of ATM, MRN, and thus the homologous recombination repair signaling, which in turn will trigger chemotherapeutic resistance in patients. We noticed the sensitivity of PRDX1-deleted cells to cisplatin, daunorubicin, and ATM inhibitors, and this sensitivity was significantly enhanced in combination with a low dose of arsenite, which caused the rapid degradation of key homologous recombination DNA repair proteins. Thus, it is predicted that arsenite will sensitize tumours to many other chemotherapeutic agents that act by damaging the DNA and more profoundly if the cancer cells are deficient in PRDX1 function [12]. As such, we propose that small molecule inhibitors of PRDX1, or single nucleotide polymorphisms that compromise PRDX1 function, in combination with low doses of arsenite can be exploited to treat chemo-resistant tumours.

## MATERIALS AND METHODS

Arsenite and N-ethyl maleimide (NEM) were obtained from Sigma-Aldrich. ATM inhibitors AZ31 and KU55933 were a gift from AstraZeneca. Cisplatin and Daunorubicin were a gift from Prof. S. Madhusudan Lab, UK.

### Cell culture and generation of PRDX1 knockouts

HEK293T and HeLa cell lines were obtained from the American Type Culture Collection (ATCC, VA, USA). Cells were propagated in Dulbecco’s modified Eagle’s medium (DMEM) supplemented with 10% fetal bovine serum (FBS) and 1% Penicillin streptomycin. PRDX1 knock-out in HeLa cells was done by infecting cells with lentivirus encoding shRNA targeting.

PRDX1(TGCTGTTGACAGTGAGCGACCAGATGGTCAGTTTAAAGATTAGTGAAGCCACAGATGTAATCTTTAAACTGACCATCTGGCTGCCTACTGCCTCGGA), or a scrambled (SCR) shRNA. Stable clones were selected in 5 μg/mL puromycin [4]. PRDX1 knock-out in HEK293T cells was generated by CRISPR-Cas9 as per the protocol described in [31]. Briefly, gRNA oligonucleotides targeting PRDX1 were cloned in the px330 plasmid vector provided by Feng Zhang (Addgene plasmid # 42230). For both cell lines Positive clones were validated by Sanger sequencing.

### Clonogenic survival assay

250 Cells/well were seeded in 6-well plates and left to adhere overnight at 37°C in a 5% CO_2_ atmosphere. The following day cells were treated with the inhibitors and incubated for 14 days. For arsenite single agent treatment, cells were treated with the indicated arsenite doses diluted in PBS for 30 min. Then PBS was removed, and cells were topped up with complete culture media and left to form clones for 14 days. For arsenite, daunorubicin, or cisplatin combination, cells were treated with 5μM arsenite for 30 min, then daunorubicin or cisplatin was added at the indicated concentration. After colony formation, plates were washed with PBS, fixed, and stained with a crystal violet-methanol mixture and colonies were counted.

### MTT cell proliferation assays

Cells were plated in 96-well plates at 200 cells/well density in a complete medium and left overnight. Then cells were treated with arsenite concentrations diluted in DPBS for 30 min. PBS was removed, and cells were topped up with fresh culture media and incubated for five days. Cell viability was measured using the MTT cell viability assay reagent (Invitrogen, UK).

### Western blot

After treatment, cells were washed with 100 mM N-ethylmaleimide (NEM) in PBS for 10 min on ice to block cysteine residues and prevent post-lysis modifications. Cells were scraped and pelleted, then resuspended in lysis buffer (150 mM NaCl, 20 mM Tris-Cl pH 7.5, 0.5% NP-40, 5 mg/ml NEM, 1X protease and phosphatase inhibitor cocktail (Sigma)). Samples were sonicated in a Bioruptor sonicator on high power (5 pulses, 10 seconds on, 10 seconds off). Samples were pelleted in a microcentrifuge at 14,000 rpm for 10 min. Protein lysates were diluted with non-reducing Laemmli buffer without β-mercaptoethanol. Samples were analyzed by western blot.

### Q-PCR analysis

Cells were plated overnight in T25 flasks and treated with 100 μM or 2.5 mM arsenite in DPBS for 10 min. Cells were collected in the RLT buffer (Qiagen, Hilden, Germany) and RNA extraction was performed using the RNAeasy mini kit (Qiagen). cDNA was synthesized from the total RNA (0.5 μg) using a high-capacity cDNA reverse transcription kit (Thermofisher Scientific, Waltham, MA, USA), according to the manufacturer’s protocol. Real-time PCR (qPCR) was performed using SYBR Green Master Mix (ThermoFisher Scientific). Samples were run on Quanti Studio 6 Flex qPCR machine. GAPDH was used as a loading control.

### Protein stability assays

Cells were seeded overnight and then treated with 25 μM MG132 (Sigma) for 3 h to inhibit the proteasome machinery. After MG132 treatment, cells were treated with 100 μM or 2.5 mM arsenite in PBS for 10 min. Cells were incubated with 100 μM NEM in PBS on ice then scraped and centrifuged. Pellets were resuspended in lysis buffer containing NEM (150 mM NaCl, 20 mM Tris-Cl pH 7.5, 0.5% NP-40, 5 mg/ml NEM, 1X protease, and phosphatase inhibitor cocktail) and then analyzed by western blot.

### Co-immunoprecipitation

Cell lysates were extracted in RIPA buffer containing protease and phosphatase inhibitors cocktail on ice. Lysates were sonicated and centrifuged at 13000 RPM for 10 min at 4^°C^. Protein extracts were incubated with the target antibodies overnight and then conjugated to protein A/G magnetic beads for 2 h at room temperature. After IP the beads were washed 4 times thoroughly with Phosphate buffer saline containing 0.01% Tween 20 and protease inhibitors. Immunoprecipitated proteins were eluted using 4× SDS loading buffer and then heated at 95^°C^ for 10 mins. Samples were run on 4–12% SDS PAGE.

### Immunofluorescence

Cells were seeded on the coverslips coated with Poly-D -lysine overnight and then treated with arsenite for 10 min. Cells were fixed with 10% formalin for 20 min and permeabilized with 0.1% Triton X-100 (Thermofisher) for 30 min. Cells were blocked with 3% BSA cells for 1 hr, then incubated with anti-ATM and anti-H2AX antibodies for 16 h at 4^°C^. Cells were labeled with Goat anti-rabbit IgG Alexa fluor 488 and Goat anti-mouse IgG Alexa fluor 594 for 1 hr. Slides were prepared in duplicates. Imaging was carried out using a Leica confocal microscope. Analysis was performed in ImageJ Software.

### Cell cycle and γH2AX analysis by flow cytometry

Cells were plated overnight, then treated with arsenite in PBS for 30 min or left untreated. After treatment, cells were washed and left in fresh media for 16 h. Cells were collected by trypsinization, washed with ice-cold PBS, and fixed in 70% ethanol for at least 30 min. Cells were permeabilized with 0.01% triton-x100 and stained with phosphor-Histone (γH2AX) Ser139 for double-strand break detection. For cell cycle progression, cells were treated with RNase and stained with 10 μg/ml propidium iodide (Sigma Aldrich) in PBS. Samples were analyzed on a flow cytometer (Beckman Coulter) and data were analyzed using FlowJo software.

### Clinical study

#### Patients selection

Investigation of the expression of PRDX1 protein in ovarian epithelial cancer was carried out on tissue microarrays of 331 consecutive ovarian epithelial cancer cases treated at Nottingham University Hospitals (NUH) between 1997 and 2010. Patients were comprehensively staged as per the International Federation of Obstetricians and Gynaecologists (FIGO) Staging System for Ovarian Cancer. Platinum resistance was defined as patients who had progression during first-line platinum chemotherapy or relapse within 6 months after completion of chemotherapy. Overall survival (OS) was calculated from the operation date until the time of death or the last date of follow-up when any remaining survivors were censored. Progression-free survival was calculated from the date of the initial surgery to disease progression or from the date of the initial surgery to the last date known to be progression-free for those censored. Tumour Marker Prognostic Studies (REMARK) criteria, recommended by McShane et al. [32] were followed throughout this study. This study was carried out by the declaration of The Helsinki and ethical approval which was obtained from the Nottingham Research Ethics Committee (REC Approval Number 06/Q240/153). All patients provided informed consent.

#### Tissue Microarray (TMA) and Immunohistochemistry (IHC)

Tumour samples were arrayed in tissue microarrays (TMAs) constructed with 2 replicate 0.6 mm cores from the tumours. Immunohistochemical staining was performed using the Novolink Max Polymer Detection System (RE7280-K: 1250 tests, Buffalo Grove, IL, USA), and the Leica Bond Primary Antibody Diluent (AR9352, Buffalo Grove, IL, USA), each used according to the manufacturer’s instructions. Pre-treatment antigen retrieval was carried out on the TMA sections using citrate buffer (pH 6.0) and heated at 95^°C^ in a microwave (Whirlpool JT359 Jet Chef 1000W, UK) for 20 min. Slides were incubated with the primary antibodies; PRDX1 (Abcam clone ab41906) at a dilution of 1:1000 for 60 minutes at room temperature, anti-Mre11 mouse monoclonal antibody (clone ab214, Abcam), at a dilution of 1:800, for 1 h at room temperature or anti-ATM (clone Y170, Abcam) at a dilution of 1: 100, 18 h 4°C.

### Evaluation of immune staining

PRDX1 and Mre11 showed both nuclear and cytoplasm expression while ATM showed nuclear expression. The percentage of tumour cells in each category was estimated (0–100%). The H-score (range 0–300) was calculated by multiplying the intensity of staining and the percentage of staining. Low/high nuclear PRDX1 expression was defined by an X-tile H-score of ≤220. Low/high cytoplasmic PRDX1 expression was defined by an X-tile H-score of ≤200. A median H-score of ≤110 and ≤60 was used as the cut-off for high Mre11 nuclear and cytoplasmic expression respectively.

### Statistical analysis

Statistics were performed using SPSS, version 28.0 (Chicago, IL, USA). Association with clinical and pathological parameters using categorized data was examined using the Chi-squared test. All tests were 2-tailed. Survival rates were determined using the Kaplan–Meier method and compared by the log-rank test. A *p*-value of < 0.05 was identified as statistically significant. This work was approved by the Nottingham Research Ethics Committee.

Specificity of PRDX1 antibody in ovarian cancer tissue were validated by western blot in A2780 (platinum-sensitive) and A2780cis (platinum-resistant) ovarian cancer cells. A specific band for PRDX1 protein was observed at the predicted molecular weight (22 kDa).

## SUPPLEMENTARY MATERIALS


