# Supplementary material for: PRDX1 protects ATM from arsenite-induced proteotoxicity and maintains its stability during DNA damage signaling
Source: Oncotarget. 2025 May 19;16:362–78. doi: 10.18632/oncotarget.28720 (PMC12088036; doi:10.18632/oncotarget.28720)
Supplement: Supplementary file 1 [file oncotarget-16-28720-s001.pdf]

PRDX1 protects ATM from arsenite-induced proteotoxicity and maintains its stability during DNA damage signaling

SUPPLEMENTARY MATERIALS

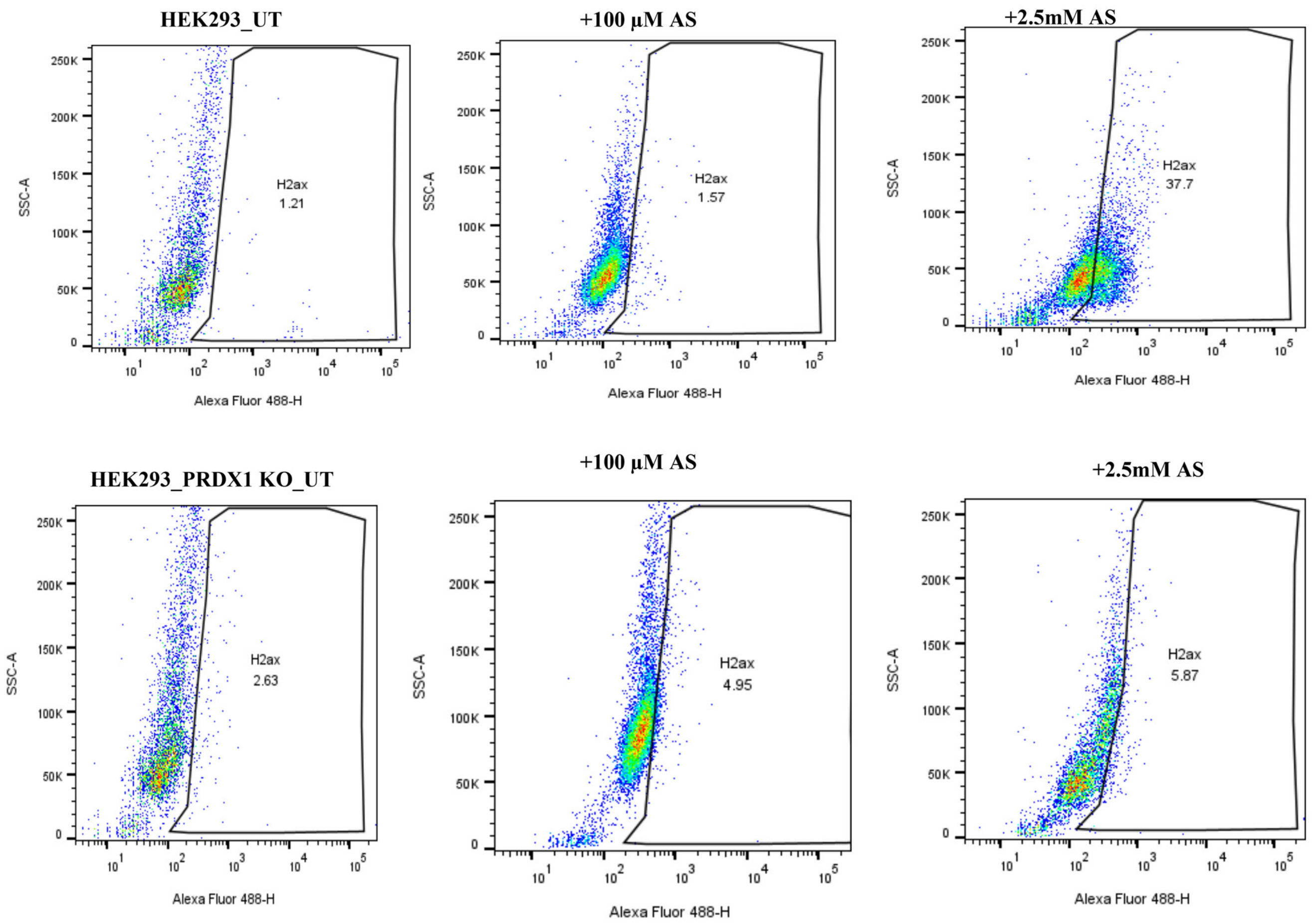

Supplementary Figure 1: Representation of the flow cytometry analysis run in FlowJo software for cell cycle exp. in HEK293 and HEK293 PRDX1 KO treated with 100 µM and 2.5 mM of arsenite.

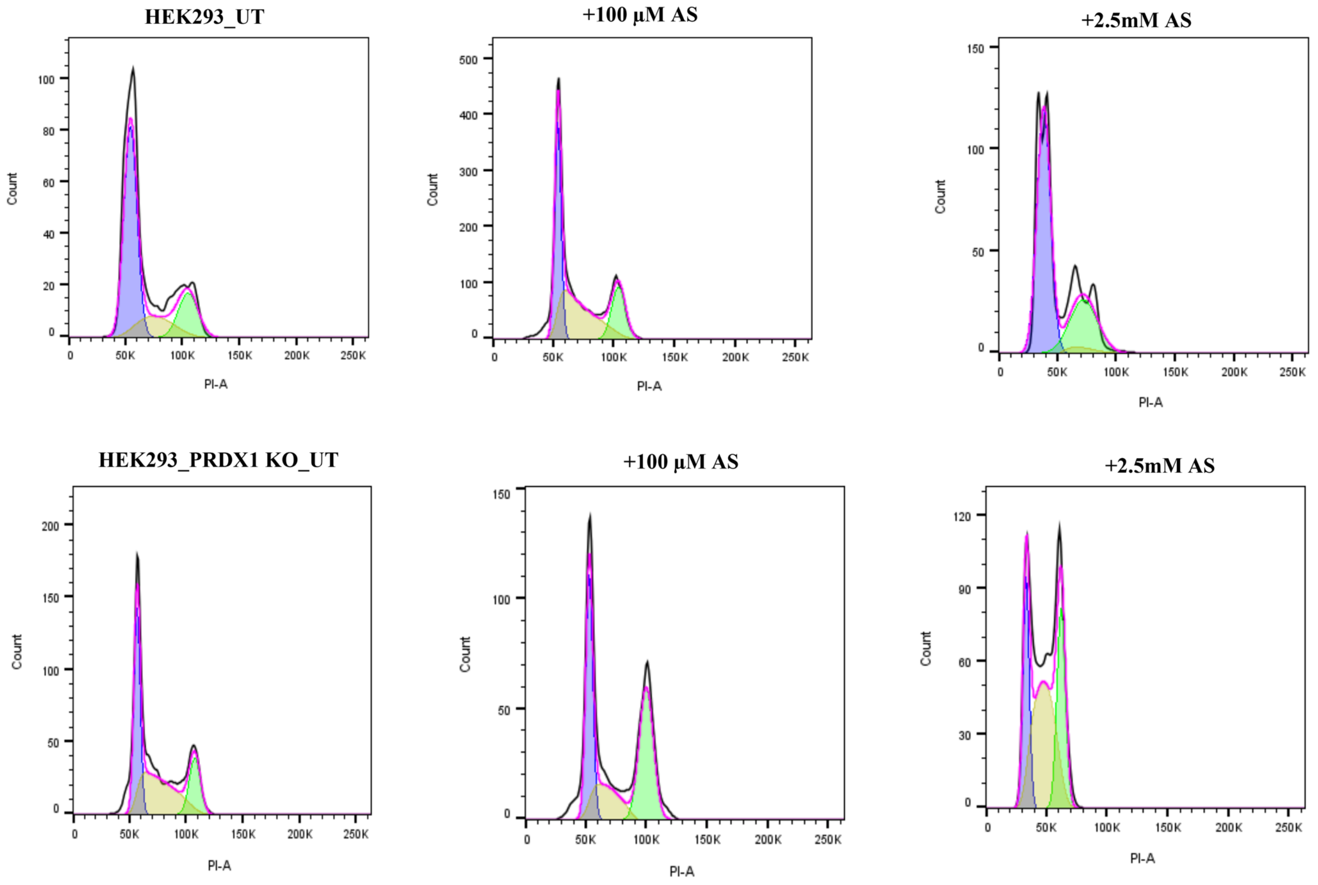

Supplementary Figure 2: Representation of the flow cytometry analysis run in FlowJo software for γH2AX exp. in HEK293 and HEK293 PRDX1 KO treated with 100 µM and 2.5 mM of arsenite

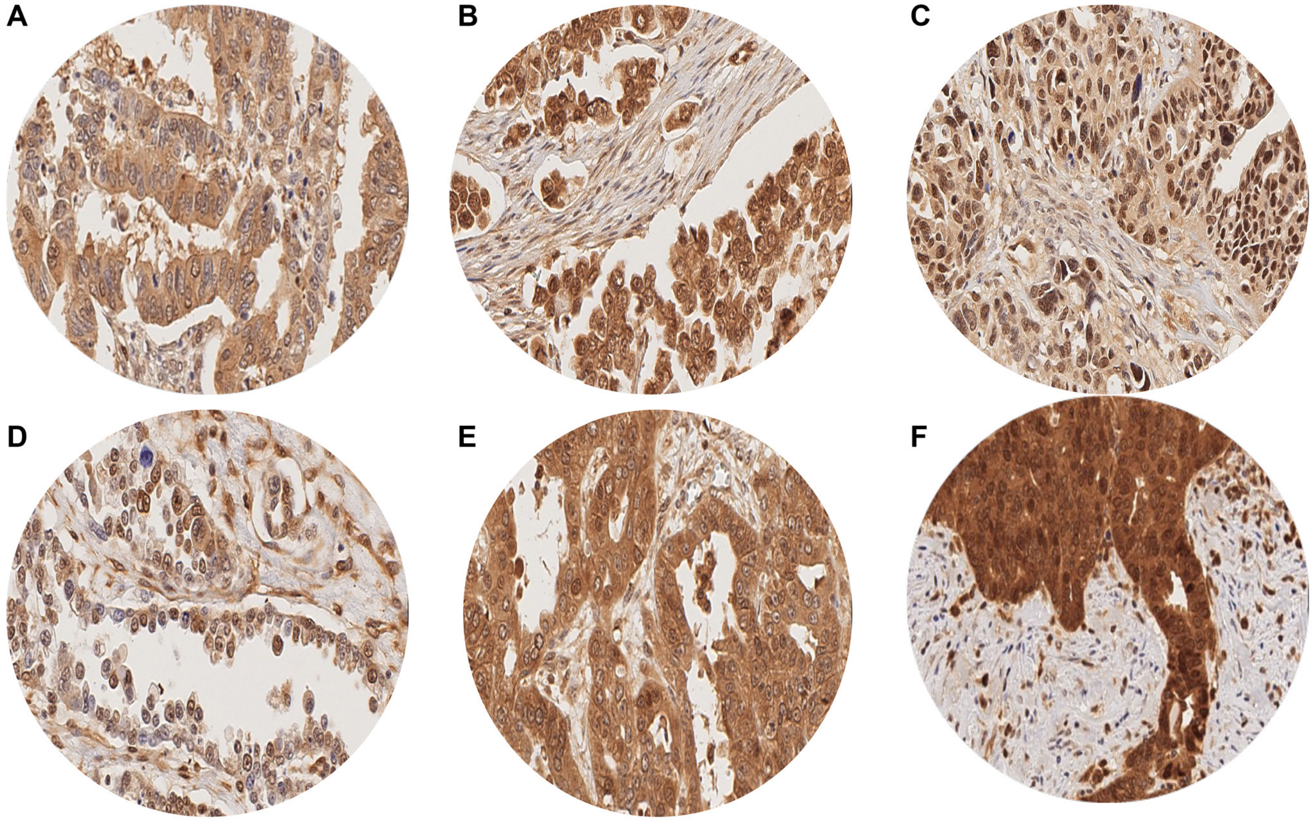

Supplementary Figure 3: Photomicrographs of PRDX1 expression in ovarian TMA. PRDX1 nuclear expression showed different staining intensities (**A**) mild, (**B**) moderate and (**C**) high. PRDX1 Cytoplasmic expression was variable (**D**) weak, (**E**) moderate and (**F**) high intensity. The scale bar in all photos is 50 µm.

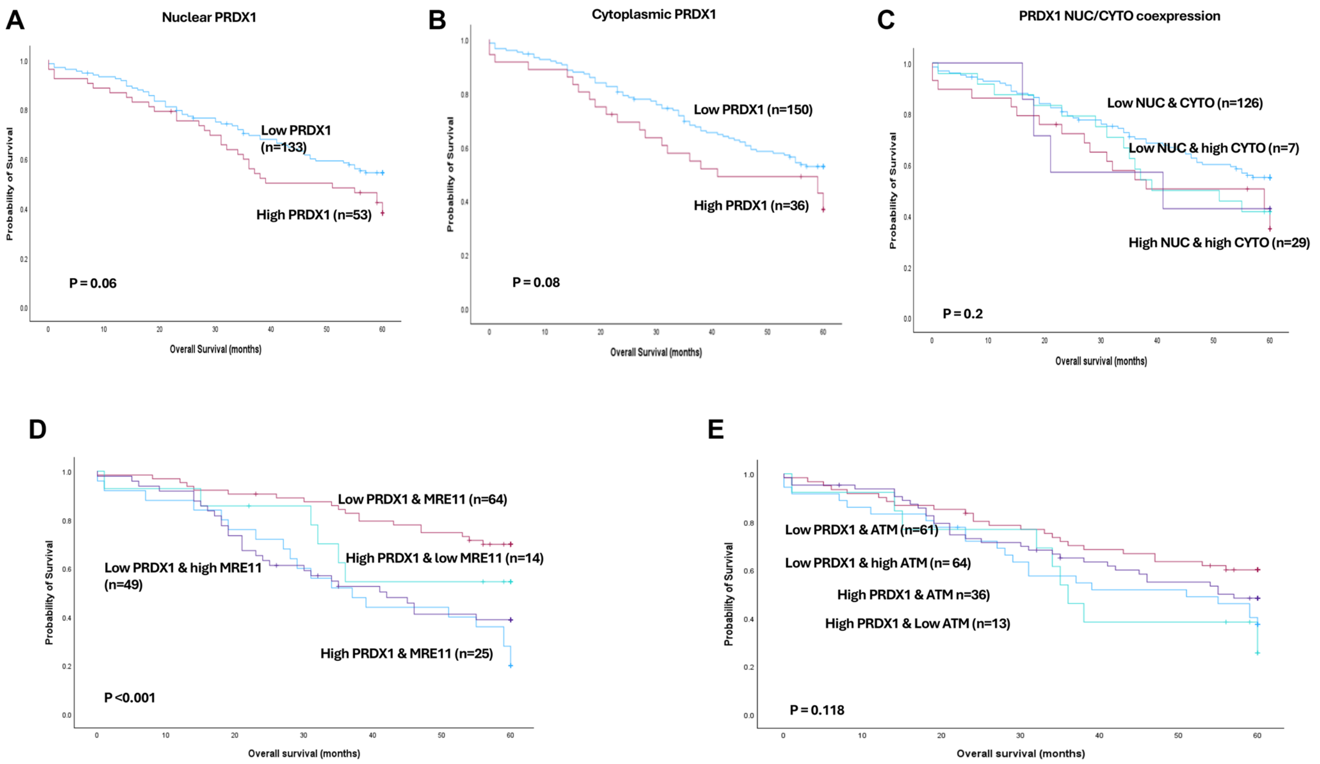

Supplementary Figure 4: Kaplan-Meier survival analysis showing survival outcome of PRDX1 in ovarian cancer. (**A**) PRDX1 nuclear expression with overall survival. (**B**) PRDX1 cytoplasmic expression with overall survival (**C**) Combined nuclear and cytoplasmic expression with overall survival. (**D**) PRDX1 and MRE11 co-expression and overall survival. (**E**) PRDX1 and ATM co-expression and overall survival
